# *Paradendryphiella arenariae* an endophytic fungus of *Centella asiatica* inhibits the bacterial pathogens of fish and shellfish

**DOI:** 10.3389/fmicb.2024.1441525

**Published:** 2024-10-31

**Authors:** Saranya Shankar, Mythili Sathiavelu

**Affiliations:** Department of Biotechnology, School of Bio Sciences and Technology, Vellore Institute of Technology, Vellore, India

**Keywords:** aquaculture, endophytic fungus, *Paradendryphiella arenariae*, bacterial fish pathogens, *Centella asiatica*, disease control, antibacterial agents

## Abstract

**Introduction:**

Aquaculture has been considered a major food-producing sector in the world during the last few decades. The foremost constraint in the development of aquaculture is bacterial disease control and management. Since various fish pathogens are resistant to conservative treatments, it is essential to screen new and effective alternative antibacterial agents. Endophytic fungi are microorganisms that live in the plant’s internal tissues without harming its host. Endophytic fungi have proven themselves as reliable sources of novel bioactive compounds that can be used as antibacterial agents.

**Methods:**

In the present study, fifteen morphologically different endophytic fungi were isolated from the fresh and healthy stem section of *Centella asiatica*. The active endophytic fungal crude extracts were tested for agar well diffusion assay, Minimum Inhibitory Concentration, Minimum Bactericidal Concentration assays, Time-kill kinetic analysis, Brine shrimp lethality assay.

**Results and Discussion:**

Agar plug diffusion and agar well diffusion assays revealed that endophytic fungus CAS1 exhibits maximum antagonistic activity against bacterial fish pathogens. The ethyl acetate crude extract of CAS1 exhibited the maximum zone of inhibitions against *Aeromonas hydrophila* (21  ±  0.11  mm), *Aeromonas caviae* (18  ±  0.1  mm), *Edwardsiella tarda* (23  ±  0.11  mm), Vibrio anguillarum (19  ±  0.05  mm) and *Vibrio harveyi* (20  ±  0.27  mm). The MIC and MBC values extract varied reliant on the trial pathogens ranging between 12.5-100 μg/mL and 25-100μg/mL correspondingly. The morphological and molecular characterization of potential isolate CAS1 was confirmed as *Paradendryphiella arenariae* by 18S rRNA ITS gene sequencing with 99.18% identity. This is the foremost findings to study the antagonistic effect of *Paradendryphiella arenariae* isolated from the stem of *Centella asiatica* against bacterial fish pathogens which can be used as natural effective antibacterial agents in aquaculture.

## 1 Introduction

The aquaculture sector plays a significant food security role in developing countries by subsidizing both domestic and world food supply development (Nabila and Kannabiran, 2018). Globally, around 950 million people take fish as the crucial source of animal protein (16%), minerals, micronutrients and omega-3—3 fatty acids in their diets. India is considered one of the leading countries worldwide by producing over 13 lakh tonnes of aquaculture (Pradeepkiran, 2019). Fish disease is one of the most important hindrances to the development of the aquaculture trade due to stress factors that cause loss of millions of dollars every year. Microorganisms are the causative agent of infectious diseases in aquaculture. Bacterial fish diseases are responsible for the main serious problem in aquaculture management (Selvaraj et al., 2014). Bacterial fish diseases such as Motile *Aeromonas* septicemia (MAS), Hemorrhagic septicemia, Epizootic ulcerative syndrome, Edwardsiellosis, and Vibriosis, etc., cause severe disease outbreaks in various species of fish that are accountable for economic downfall in aquaculture production (Irshath et al., 2023). *Aeromonas* sp. in aquaculture is considered a common disease-causing pathogen that results in substantial fish mortality and huge economic losses (Leão et al., 2020). *Edwardsiella tarda* is an intracellular pathogen that produces infections in a huge number of hosts like fishes, birds, amphibians, mammals and reptiles (Xu and Zhang, 2014). Most of the economically important fishes present in the marine and aquatic zones are affected by halophilic bacteria including *Vibrio anguillarum* and *Vibrio harveyi*. The vaccines produced cannot be used for controlling the diseases because they are massive expensive labor and extremely time-exhaustive process in aquaculture. In recent years, antibiotics have been widely used in the aquaculture industry for disease control but the frequent use of these antibiotics as antibacterial agents has led to the development of antibiotic resistance (Nabila and Kannabiran, 2018). To combat all these problems, the search for extremely active, safe, novel bioactive compounds from natural sources to fight against bacterial fish pathogens remains a higher priority.

Natural products and secondary metabolites are known as chemical substances that have been extracted from various living organisms. Nowadays, drugs from numerous significant bioactive compounds are isolated from plants (Nisa et al., 2022). Endophytes are a group of microbes that colonize a portion of the living plant’s internal tissues without harming its host (Maheswari and Saranya, 2018). Several studies have stated that endophytic fungi are abundant and they can be attained in all plant species (Huang et al., 2007). Endophytic fungi are capable of producing an extensive diversity of bioactive secondary metabolites with distinctive structures including flavonoids, phenolic acids, alkaloids, benzopyranones and quinines. Such active metabolic substances are utilized as immunosuppressants, antimicrobials, antioxidants, antibiotics, agrochemical, and anticancer agents (Fernandes et al., 2015; Nath et al., 2014).

An ethno- medicinal plant *Centella asiatica* is a small, slender, annual, prostrate herb with several branches belonging to the family Apiaceae. It is generally referred to as Indian pennywort or Gotu kola and is primarily found in tropical areas of countries like India, China, Australia, Madagascar, Indonesia, Sri Lanka, and South Africa (Shastry et al., 2020; Rakotoniriana et al., 2008). *Centella asiatica* is an odorless and intense bitter taste plant. The leaves of the plant are small and are fan-shaped green in color with light purple-to-pink or white flowers and produce oval-shaped fruit (Gohil et al., 2010). The most important chemical constituents that are found in *C.asiatica* are asiaticoside, asiatic acid, terpenoids, madecassoide, stigma sterol, madecassic acid, glucose, sitosterol, rhamnose, vitamins and fatty oils. It is also useful in the prevention of several diseases such as psoriasis, leprosy, tuberculosis, diarrhea, lupus and asthma, etc., (Nath et al., 2014). The latest study reported that the secondary metabolites like Azaanthraquinones and Napthaquinones derived from endophyte *Fusarium solani* ethyl acetate extract of the medicinal plant *Centella asiatica* exhibited significant anticancer and antibacterial action (Moni et al., 2022). Jalil et al. (2022) reported that fungal endophyte *L. pseudotheobromae* possesses significant antagonistic activity against *E. profundum* and *V. parahaemolyticus*. Thus the current study aims to isolate and identify the endophytic fungi from *Centella asiatica* to investigate for their antibacterial efficacy on bacterial fish pathogens.

## 2 Materials and methods

### 2.1 Plant sampling

The Fresh and healthy stems of *Centella asiatica* were collected from Vellore (12°90′00.0” N 79°15′90.0″ E), Tamil Nadu, India in a sterile polythene bag and transported to the laboratory for the endophytic fungi isolation. Taxonomically, the plant was authenticated by Dr. S. Geetha, Head and Assistant Professor, Department of Botany, AAGA College for Women, Walajapet—632,513.

### 2.2 Surface sterilization of plant for endophytic fungi isolation

The endophytic fungi were isolated from stem sample of *Centella asiatica* using a standard protocol with slight modifications (Prabakaran et al., 2012). The stem part from *Centella asiatica* was carefully detached and rinsed in water to remove the adhered dust and debris. It was surface disinfected by soaking them in a solution of 5% sodium hypochlorite for 2–3 min following 1 min in 70% ethanol. Finally, samples were washed three times with Milli Q and dried with blotting paper. Then it was cut into small segments (0.5 cm) and impregnated on the Potato dextrose agar (PDA) medium (Hi-Media) and incubated at 28°C for 1 week for the isolation of endophytic fungi. Then the evolving fungus was transferred and the pure cultures were preserved in PDA slants at 4°C. To examine the efficiency of surface sterilization, the last rinsed (1 mL) milli Q was spread on the PDA plate.

### 2.3 Test pathogens and culture standardization

Pathogenic bacteria in aquaculture such as *Aeromonas caviae* (MTCC 7725), *Aeromonas hydrophila* (MTCC 1739), *Edwardsiella tarda* (MTCC 2400), *Vibrio anguillarum* (ATCC 43305) and *Vibrio harveyi* (MTCC 7954) were used for the study. The bacterial cultures *Aeromonas caviae*, *Aeromonas hydrophila* and *Edwardsiella tarda* were grown on a nutrient agar medium. On the other hand, *Vibrio anguillarum* and *Vibrio harveyi* were grown on nutrient agar medium supplemented with 2% NaCl and incubated at 37°C for 24 h. The test pathogens were stored at −20°C in nutrient broth with glycerol stock. The bacterial inoculums were standardized with Mc Farland (0.5) to get approximately 1 × 10^8^ CFU/mL cell suspensions.

### 2.4 Initial screening of endophytic fungi

#### 2.4.1 Agar plug diffusion test

The initial assessment of the isolates were studied by agar plug diffusion assay to determine the hostile activity according to the procedure mentioned by Zerroug and Sadrati (2022) with slight modifications. To obtain agar plugs (6 mm in diameter), the fungal isolates were grown on PDA plates and incubated at 28°C for 5–7 days before plugging by sterile cork borer. Then the plugs were transferred onto the Mueller Hinton Agar (MHA from Hi-Media) medium spread with the trial microorganisms. Plates were then sealed using parafilm and primarily kept overnight refrigeration at 4°C to facilitate bioactive compounds diffusion and then incubated at 37°C for 12–18 h. The inhibition zone around the plugs was observed. The experiment was performed in triplicates.

### 2.5 Effect of fungal crude extract against bacterial fish pathogens

#### 2.5.1 Fermentation and extraction of endophytic fungal crude extracts

The grown pure mycelial agar plugs from the PDA plate were transferred into a 500 mL flask (Borosil) containing 300 mL of potato dextrose broth (PDB purchased from Hi-Media) and incubated at 28°C for 21 days. Post incubation, the fermented broth of each isolate was separated from mycelium using Whatman No. 1 filter paper. Based on different polarity, the filtrate was extracted with solvents acquired from SRL such as petroleum ether (Pet ether), dichloromethane (DCM), ethyl acetate (EtOAc), and butanol by utilizing a separating funnel (Borosil) to get fungal crude secondary metabolites. The organic phase was evaporated and the obtained fungal crude extracts were dried. The crude extract stock solution (1 mg/mL) was prepared by dissolving it in DMSO (Dimethyl sulphoxide) and used to determine the antibacterial activity (Devi and Prabakaran, 2014).

#### 2.5.2 Antagonistic activity by agar well diffusion assay

The endophytic fungal isolates that showed higher antagonist action in initial screening were subjected to secondary analysis. The agar well diffusion technique was performed to determine the antibacterial property (Avinash et al., 2015). Different trial organisms were uniformly streaked over the surface of the freshly prepared MHA dish using sterilized swabs. The wells (5 mm) were punched on the Petri dish by a sterile cork borer. Then the respective well was poured with 100 μL of fungal crude extract at different concentrations (25, 50 and 100 μg/mL). Ciprofloxacin disk (5mcg) serves as positive and well-containing DMSO (5%) was considered a negative control. At 37°C, the inoculated Petri dishes were incubated for 24 h. After the overnight incubation period, the inhibition zone was calculated in millimeters (mm). The test was done in triplicates.

### 2.6 Macroscopic and microscopic characterization

Pure fungal endophytes were identified by microscopic studies using Lactophenol cotton blue staining for the observation of fungal morphology and also macroscopic characterization based on their pigmentation, characteristics of the spores, aerial mycelium, texture of the surface and colony or hyphal morphology using different standard manuals (Liu et al., 2019).

### 2.7 Molecular genomic analysis of endophytic fungus

The species of the potent isolate was molecularly identified by utilizing 18S rRNA ITS sequencing. Genomic DNA was retrieved from pure fungus following the protocol illustrated by González-Teuber et al. (2017) with slight modifications. Genomic DNA was amplified using ITS1-F-KYO1 (CTHGGTCATTTAGAGGAASTAA) forward primer and ITS4 (TCCTCCGCTTATTGATATGC) reverse primer to determine the fungal endophyte species. PCR reaction mixtures (50 mL) containing 7 μL of total fungal genomic DNA, 1 μL of each primer (10 μM), 27.5 μL of SapphireAmp Fast PCR Master Mix (Takara) and 13.5 μL of sterilized water were used to conduct the amplification of ITS regions. Using Techne TC-5000 Thermal Cycler, the Polymerase chain reaction was done. The program includes 35 cycles of 1 min denaturation (94°C), 30 s of annealing (54°C), 1 min primer extension (72°C) and 7 min of final extension (72°C). The resulting sequences were used at the NCBI for BLAST search. Using version 7.0 MEGA software, the phylogenetic tree was created by the neighbor-joining tree technique.

### 2.8 Minimum inhibitory and minimum bactericidal concentration

In a sterile 96-well micro-titer plate, the two-fold broth microdilution method was performed by the procedure described by CLSI guidelines to evaluate the bactericidal and bacteriostatic of endophytic fungal ethyl acetate crude extract. The stock solution of active extract was prepared at a concentration of 1 mg/mL in 10% DMSO and 100 μL of the sample was introduced to the first well. Following, different concentrations of (100 to 0.78 μg/mL) to obtain two-fold microdilution. A volume of 100 μL MHB was filled in all the wells. To each well, 10 μL of trial cultures were added. MHB and MHB with bacteria served as positive and negative control, respectively. Post incubation at 37°C for 24 h, the lowest concentration of ethyl acetate crude extract at which no visible growth of bacteria was calculated as MIC. The dilution showed no visible bacterial growth, samples (50 μL) from the wells were streaked on the freshly prepared MHA medium to determine the MBC value and the plates were incubated at 37°C for 24 h. The lowermost dilution with complete inhibition of test microorganisms (99.9%) was determined as the MBC values. The test was done in triplicates (Valle et al., 2016).

### 2.9 MIC index of the crude extract

MIC Index was calculated to estimate the effectiveness of endophytic fungal extract whether it is bactericidal or bacteriostatic. The MIC Index of the fungal extract was computed by dividing the value of MBC by the value of MIC (Sadrati et al., 2020).

### 2.10 Time-kill kinetic assay

A time-kill assay was carried out to investigate the effectiveness of the crude against the trial pathogens according to the protocol of Rani et al. (2017). In a 50 mL Erlenmeyer flask add 900 μL of MHB and 1 mL of extract that was prepared at the final concentrations of ½ × MIC, MIC, and 2 × MIC. About 10 μL of bacterial inoculum (1 × 10^8^ CFU/mL) was subjected to the flasks and kept in a shaker at 150 rpm for 24 h. Bacterial suspension without fungal extract was used as a positive control. A volume of 25 μL of the mixture was taken at 0, 4, 8, 12, 16, 20, 28, 32, 36, 40 and 48^th^-hour intervals and spread on freshly prepared MHA medium. The plates were incubated at 37°C for 24 h. Post incubation period, the number of CFU (colony-forming units) per mL was counted. The Reduction percentage was determined by,


$$\begin{array}{l} \mathrm{Percentage reduction}=\mathrm{Initial count}\\ -\mathrm{Count}\;\mathrm{at}\;x\;\mathrm{interval}/\mathrm{Initial count}*100 \end{array}$$

### 2.11 Brine shrimp lethality assay

About 0.1 g of brine shrimp eggs (*Artemia salina* cysts) were permitted to hatch in seawater at 25°C for 48 h with continuous light and aeration. The ethyl acetate extract was prepared at 1 mg/mL concentration using seawater with different concentrations (125,100 and 50 μg/mL). Each 10 alive nauplii was transferred into a sterile plate containing test solutions and seawater (5 mL). Ten nauplii were transported to a sterile plate having 5 mL of seawater without test samples and were used as a negative control. In each sterile plate, the number of alive nauplii was counted after 4 h of incubation using a magnifying lens. The percentage of mortality of brine shrimp nauplii was calculated (Kumar et al., 2024).

### 2.12 Scanning electron microscopy

The SEM samples were prepared by the protocol of Jalil et al. (2022) to examine the spore morphology and detrimental changes of bacterial cells. In a 50 mL Erlenmeyer flask containing 8.9 mL of MHB and 1 mL of extract that was prepared at the dilution of MIC. About 100 μL of suspension (1 × 10^8^ CFU/mL) was added and kept in a shaker at 150 rpm for 48 h then samples were prepared and observed under SEM (EVO/18 Research, Carl Zeiss).

### 2.13 Phytoconstituents screening

Phytochemical analysis of the crude ethyl acetate extract was performed to determine the presence or absence of secondary metabolites such as steroids, phenols, alkaloids, terpenoids, flavonoids, tannins and saponins using standard protocol (Bhardwaj et al., 2015).

### 2.14 GC–MS analysis

The potent isolate CAS1 was subjected to GC–MS screening to identify different active substances. The instruments used for analysis are the Perkin Elmer GC model (30 m × 0.25 mm × 0.25 μm) and Clarus 680 (Mass spectrometer Clarus 600 EI). Helium served as a carrier gas with a 1 mL per minute flow rate. The initial temperature of the oven was sustained for 2 min at 60°C, ramp program was 10°C/min - 300°C for 4 min. About 1 μL of the sample was introduced in the split ratio of 10:1 whereas, the injector temperature was at 300°C. Using electron energy (70 eV) and a 50–600 m/z scan range, the ions were executed (Chakraborthy et al., 2016).

### 2.15 Spectral analysis of the fungal extract

FT-IR analysis was used to identify the types of functional groups (Chemical bonds) existing in the ethyl acetate crude extract of CAS1 using the Shimadzu IR Affinity FTIR Spectroscope. The dried fungal crude extract was used for FT-IR analysis. The infrared transmittance data was collected by FT-IR spectroscopy ranging from 4,000 to 400 cm^−1^. The chemical bonds present in the sample absorb a particular wavelength of light as viewed in the annotated spectrum. The chemical groups in a sample were read by the IR absorption spectrum (Sriram et al., 2020).

### 2.16 Statistical test

The studied results were described as means ± standard deviation. The statistics were computed by GraphPad Prism (Version 9.5.1) software.

## 3 Results

### 3.1 Isolation of endophytic fungi from *Centella asiatica*

**Figure 1:**
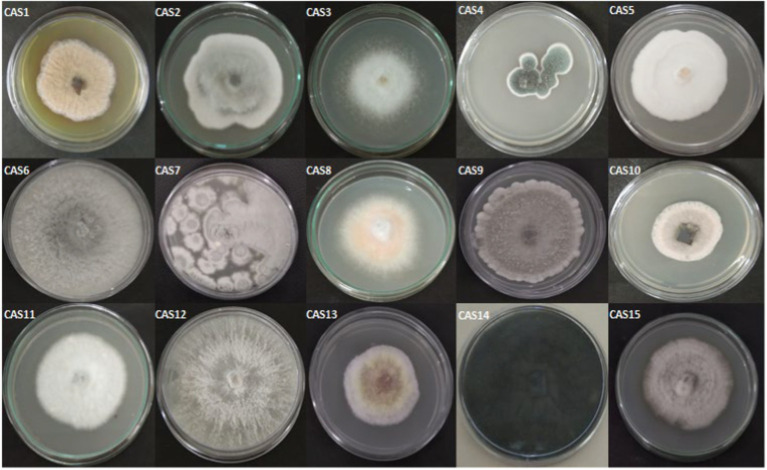
The pure isolates of endophytic fungi from *Centella asiatica* stem.

In the present study, 15 morphologically different endophytic fungi were isolated from the fresh and healthy stem section of *Centella asiatica* without any observation of bacterial and fungal growth on the PDA plates. The isolated endophytic fungi designated as CAS1-CAS15 are seen in Figure 1.

### 3.2 The preliminary antagonistic screening of endophytic fungus against bacterial fish pathogens

#### 3.2.1 Agar plug diffusion assay

**Figure 2:**
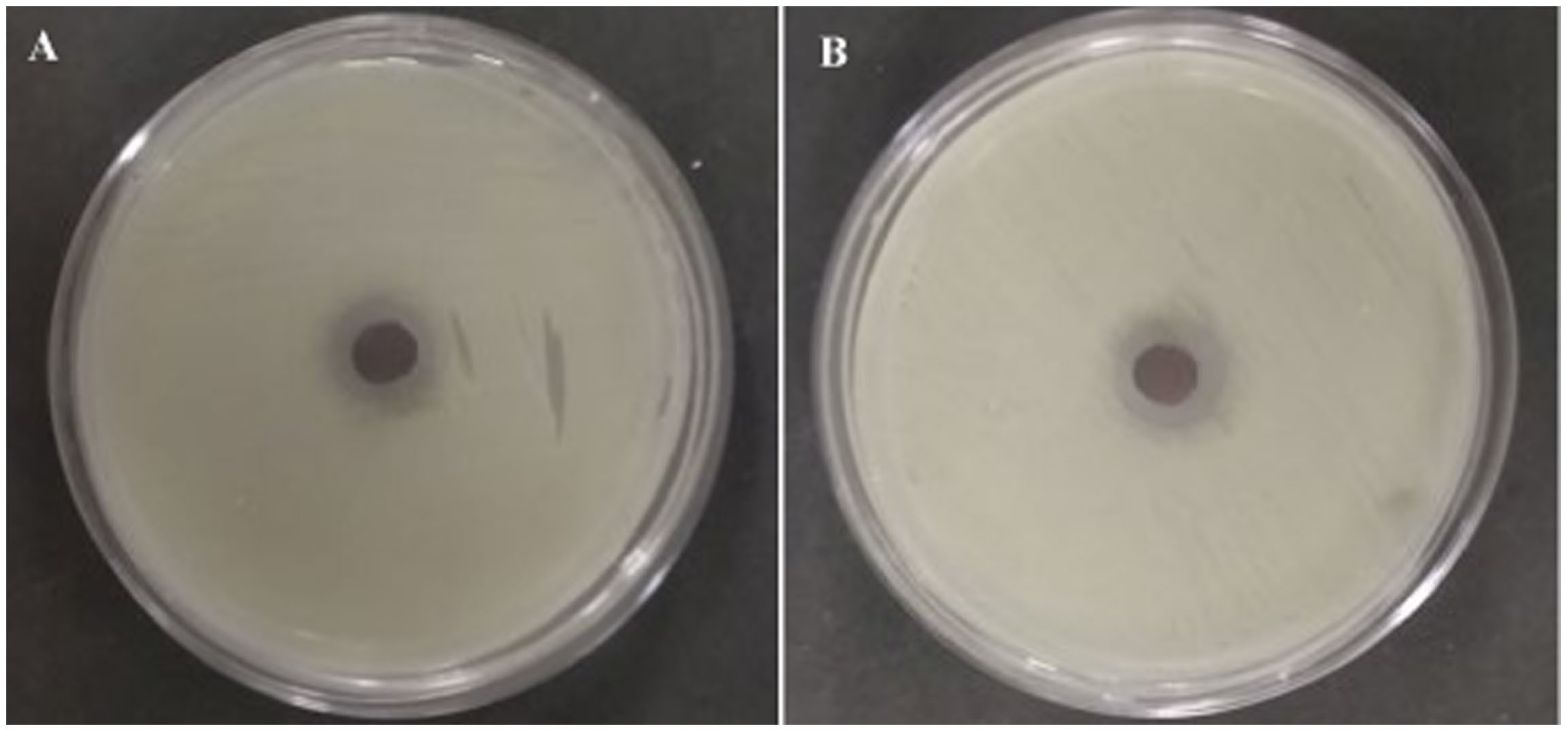
Most active endophytic fungus CAS1 isolate in Agar plug diffusion assay against **(A)** *Aeromonas hydrophila* and **(B)** *Edwardsiella tarda.*

All the 15 endophytic fungi cultured on the PDA plates were screened for antagonistic efficacy against the selected bacterial fish pathogens in the primary agar plug diffusion assay. Among the 15 isolated cultures, only CAS1 exhibits active antibacterial activity against all the selected trial pathogens are presented in Table 1. Whereas, isolate CAS14 showed the least antagonistic efficacy against *Edwardsiella tarda*. The most active of the CAS1 isolates against trial organisms are shown in Figure 2.

**Table 1:** Active endophytic fungal isolates of *Centella asiatica* on bacterial fish pathogens.

Endophytic fungal isolates	The presence of an inhibition zone				
	*A. hydrophila*	*A. caviae*	*E. tarda*	*V. anguillarum*	*V. harveyi*
CAS1	+++	+	+++	+	++
CAS14	−	−	+	−	−

“−” denotes no antibacterial activity.

### 3.3 Fermentation and extract preparation of endophytic fungus

The endophytic fungus CAS1 that displayed a broad range of antibacterial activity in primary screening was cultivated in PDB and incubated at 28°C for 21 days. Post incubation, the broth was filtered and extracted using different polarity solvents. The crude extracts were dried and used for further analysis.

### 3.4 Antagonistic activity of endophytic fungal crude extracts on test pathogens

**Figure 3:**
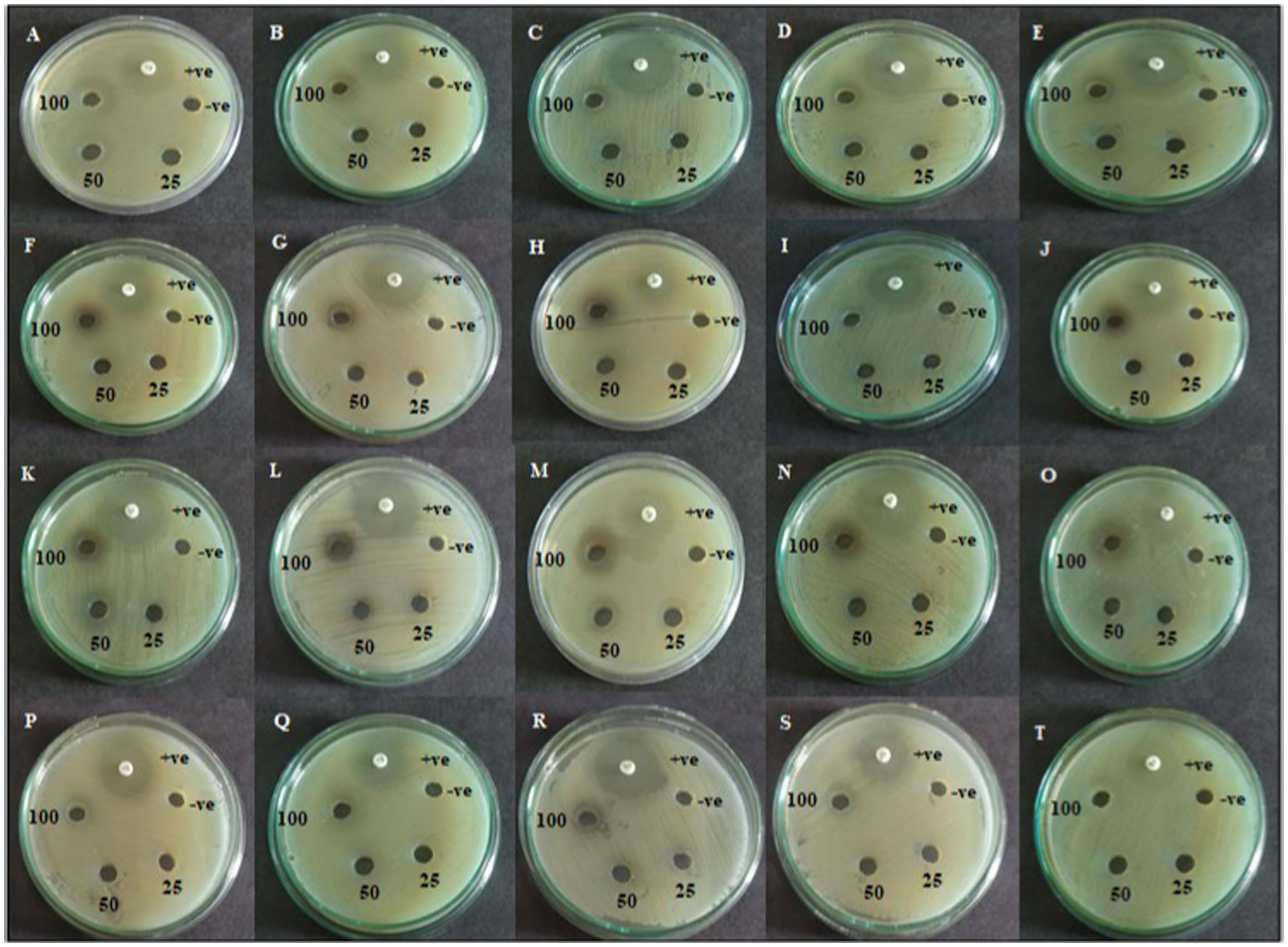
Agar well diffusion assay of active CAS1 isolate of Pet ether extract **(A–E)**; DCM extract **(F–J)**; EtOAc extract **(K–O)**; Butanol extract **(P–T)** against **(A,F,K,P)** *Aeromonas hydrophila*; **(B,G,L,Q)** *Aeromonas caviae*; **(C,H,M,R**) *Edwardsiella tarda*; **(D,I,N,S)** *Vibrio anguillarum* **(E,J,O,T)** *Vibrio harveyi.*

The agar well diffusion assay was employed to evaluate the antagonistic action against trial bacterial fish pathogens to various concentrations of resulting extracts. CAS1 crude was significantly active against aquaculture bacterial pathogens are presented in Table 2. The maximum antibacterial activity was observed in EtOAc crude extract of CAS1 isolates against *A.hydrophila* and *E.tarda* with the zone of inhibitions of 21 ± 0.11 mm and 23 ± 0.11 mm, respectively. Whereas, it showed significant antagonistic activity against *A.caviae* (18 ± 0.1 mm), *V.anguillarum* (19 ± 0.05 mm) and *V.harveyi* (20 ± 0.27 mm) inhibition zone at 100 μg/mL concentration. On the other hand, pet ether and DCM extracts exhibited antibacterial activity toward only four bacteria tested. Butanol extract possesses antibacterial activity against MTCC 1739, MTCC 2400 and ATCC 43305. Further, No zone of inhibitions was observed in the negative control (DMSO). The commercially available ciprofloxacin antibiotics were tested against these pathogens and the results are shown in Figure 3.

**Table 2:** Antagonistic activity of various crude extracts of CAS1 isolate against bacterial pathogens on agar well diffusion assay.

Extract	Concentration 100 μg/mL; Diameter of inhibition zone (mm)				
	*A. hydrophila* (MTCC 1739)	*A. caviae* (MTCC 7725)	*E. tarda* (MTCC 2400)	*V. anguillarum* (ATCC 43305)	*V. harveyi* (MTCC 7954)
Petroleum ether	19 ± 0.14	–	12 ± 0.2	12 ± 0.2	13 ± 0.05
Dichloromethane	18 ± 0.05	15 ± 0.05	16 ± 0.05	–	16 ± 0.02
Ethyl acetate	21 ± 0.11	18 ± 0.1	23 ± 0.11	19 ± 0.05	20 ± 0.27
Butanol	12 ± 0.2	–	14 ± 0.05	11 ± 0.02	–
Standard	23 ± 0.48	21 ± 0.05	27 ± 0.02	20 ± 0.25	24 ± 0.14

“−” indicates no antibacterial activity.

### 3.5 Identification of the active endophytic fungus

**Figure 4:**
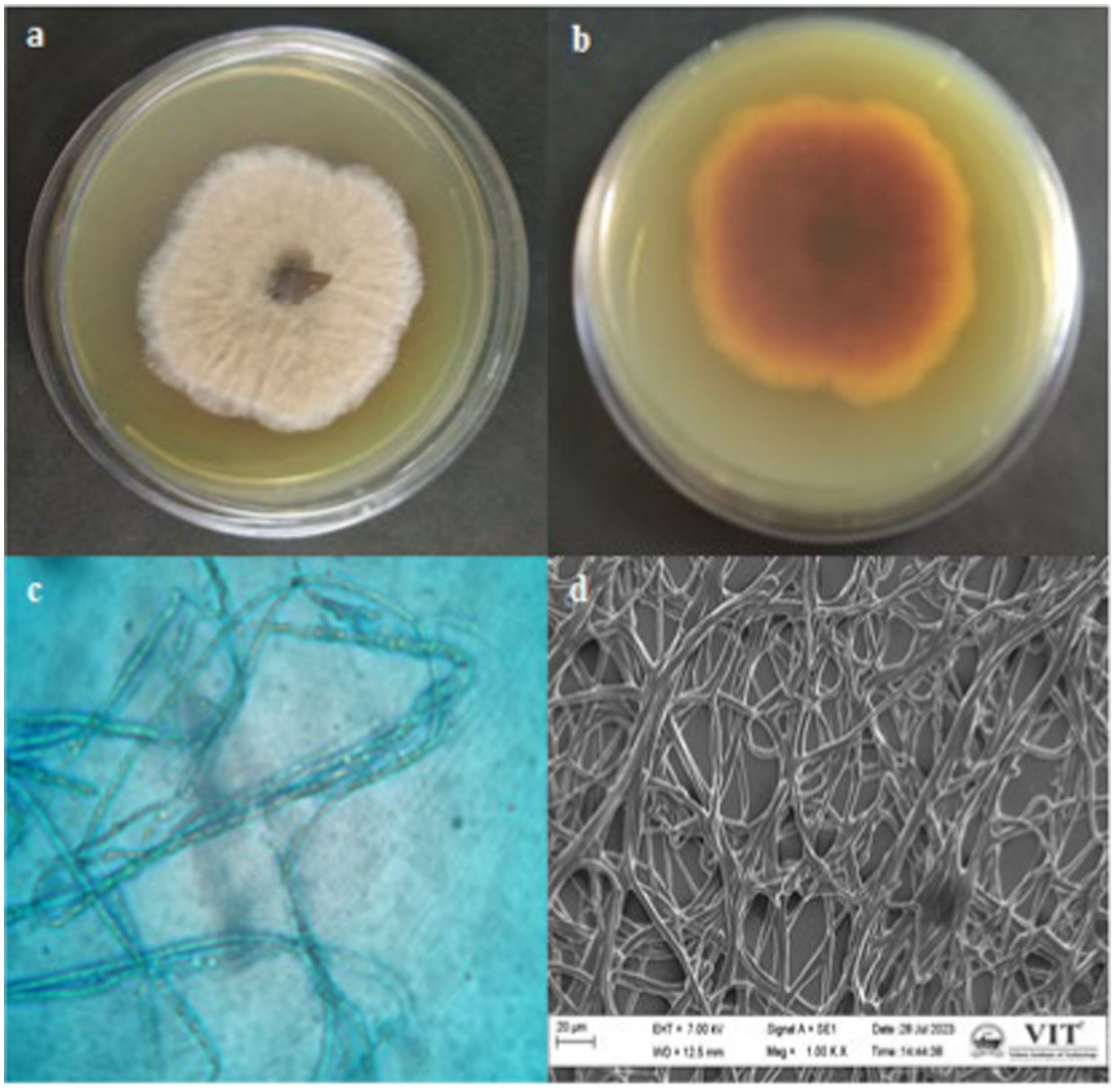
Macroscopic, Microscopic and SEM morphology of isolate from *Centella asiatica* stem **(a,b)** Growth of endophytic fungus CAS1 on PDA medium **(c)** Lactophenol cotton blue staining of CAS isolate using Light microscope with 100X magnification **(d)** SEM morphology of CAS1 isolate at 1.00KX magnification.

**Figure 5:**
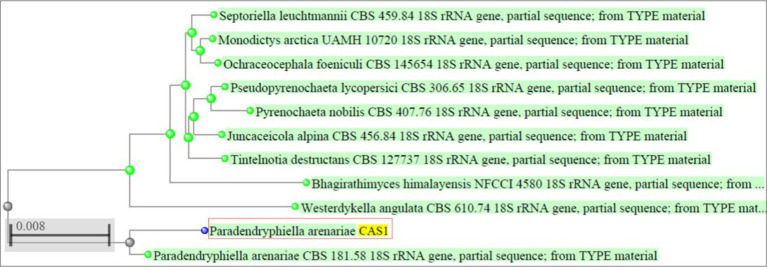
Phylogenetic tree indicating *Paradendryphiella arenariae* (CAS1) isolated from *Centella asiatica* stem based on the ITS sequences.

Based on the antibacterial effects of all the extracts against trial pathogens, the significantly active endophytic fungus CAS1 was identified morphologically and molecularly. The CAS1 isolate was a soft, orange fungus with a white edge, Orangish brown on reverse on PDA medium are shown in Figures 4a,b. The hyphae of the endophytic fungus were observed using the Lactophenol cotton blue staining technique and SEM analysis was shown in Figures 4c,d. Using molecular technique, the potent endophytic fungus CAS1 was identified as *Paradendryphiella arenariae* by 18S rRNA ITS gene sequencing with 99.18% identity. In GenBank, the sequence was deposited with accession number PP082823 and endorsed by the neighbor-joining tree are shown in Figure 5.

### 3.6 MIC and MBC of fungal crude extract

A two-fold serial dilution test was done to access the MIC and MBC of EtOAc crude extract of *Paradendryphiella arenariae* (CAS1) that exhibited maximum antagonist activity against trial bacteria in agar well diffusion assay. The findings suggested that MIC and MBC values of the extract range between 12.5–100 μg/mL and 25–100 μg/mL, respectively. The highest MIC value 12.5 ± 0.04 μg/mL was observed in *Edwardsiella tarda* (MTCC 2400) and its MBC value was found to be 25 ± 0.01 μg/mL. Followed by, *Aeromonas hydrophila* (MTCC 1739) revealed significant MIC and MBC values of 25 ± 0.5 μg/mL and 50 ± 0.2 μg/mL, respectively, in the extract. Similarly, MIC and MBC values of 100 μg/mL were detected against *Vibrio anguillarum* (ATCC 43305) are shown in Table 3.

**Table 3:** Evaluation of MIC and MBC of the ethyl acetate crude extract against fish pathogens.

Test pathogens	MIC (μg/mL)	MBC (μg/mL)	Ratio (MBC/MIC)
*A. hydrophila* (MTCC 1739)	25 ± 0.5	50 ± 0.2	2
*A. caviae* (MTCC 7725)	50 ± 0.01	100 ± 0.05	2
*E. tarda* (MTCC 2400)	12.5 ± 0.04	25 ± 0.01	2
*V. anguillarum* (ATCC 43305)	100 ± 0.05	100 ± 0.5	1
*V. harveyi* (MTCC 7954)	50 ± 0.01	100 ± 0.04	2

MIC ratio ≤ 4 is bactericidal and > 4 is bacteriostatic.

### 3.7 Time-kill kinetic analysis

**Figure 6:**
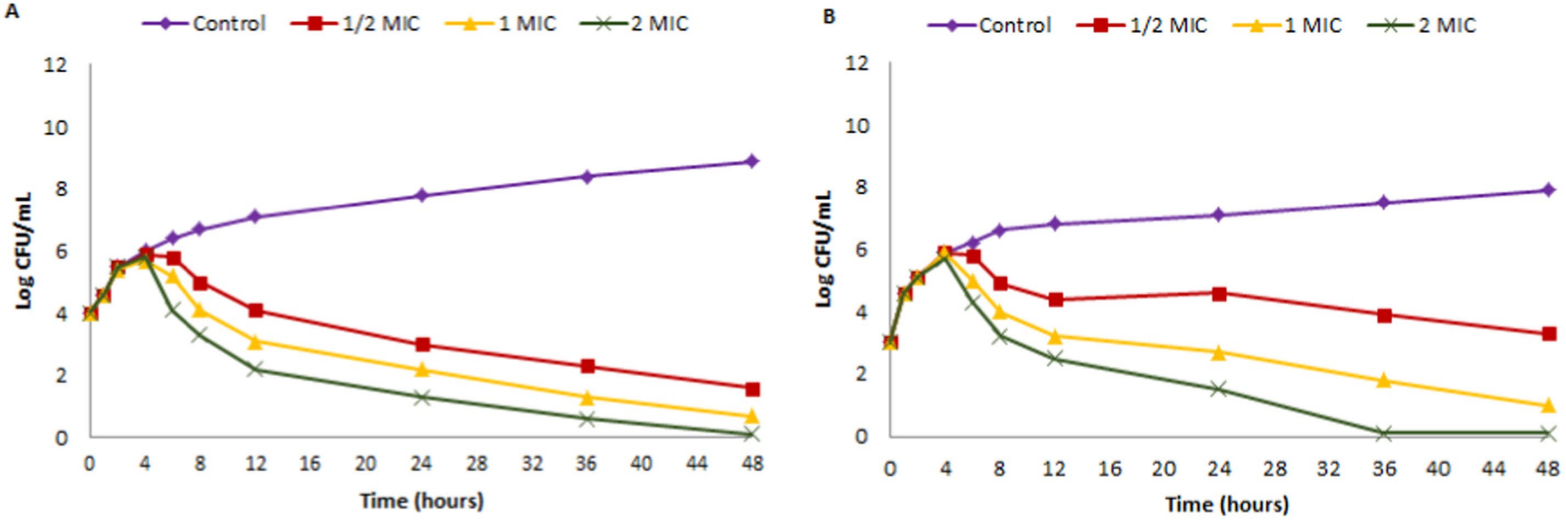
Time kill kinetics of ethyl acetate crude extract of *Paradendryphiella arenariae* against **(A)** *Aeromonas hydrophila* **(B)** *Edwardsiella tarda.*

Time kill assay was executed with bacterial fish pathogens such as *A.hydrophila* (MTCC 1739) and *E.tarda* (MTCC 2400) being exposed to ½ × MIC (12.5 μg/mL; 6.25 μg/mL), MIC (25 μg/mL; 12.5 μg/mL) and 2 × MIC (50 μg/mL; 25 μg/mL) values of EtOAc acetate crude extract of endophytic fungus *Paradendryphiella arenariae* (CAS1) over a time of 48 h. The time-kill graph (Logarithmic number of CFU/mL and time) was plotted against *A.hydrophila* and *E.tarda* and is shown in Figure 6. The results displayed a maximum reduction of bacterial cells at MIC and 2 × MIC extract concentrations post 24 h of incubation. Thus, the effect of ethyl acetate crude extract of endophytic fungus CAS1 toward *A.hydrophila* and *E.tarda* was bacteriostatic at different concentrations.

### 3.8 Brine shrimp lethality assay

**Figure 7:**
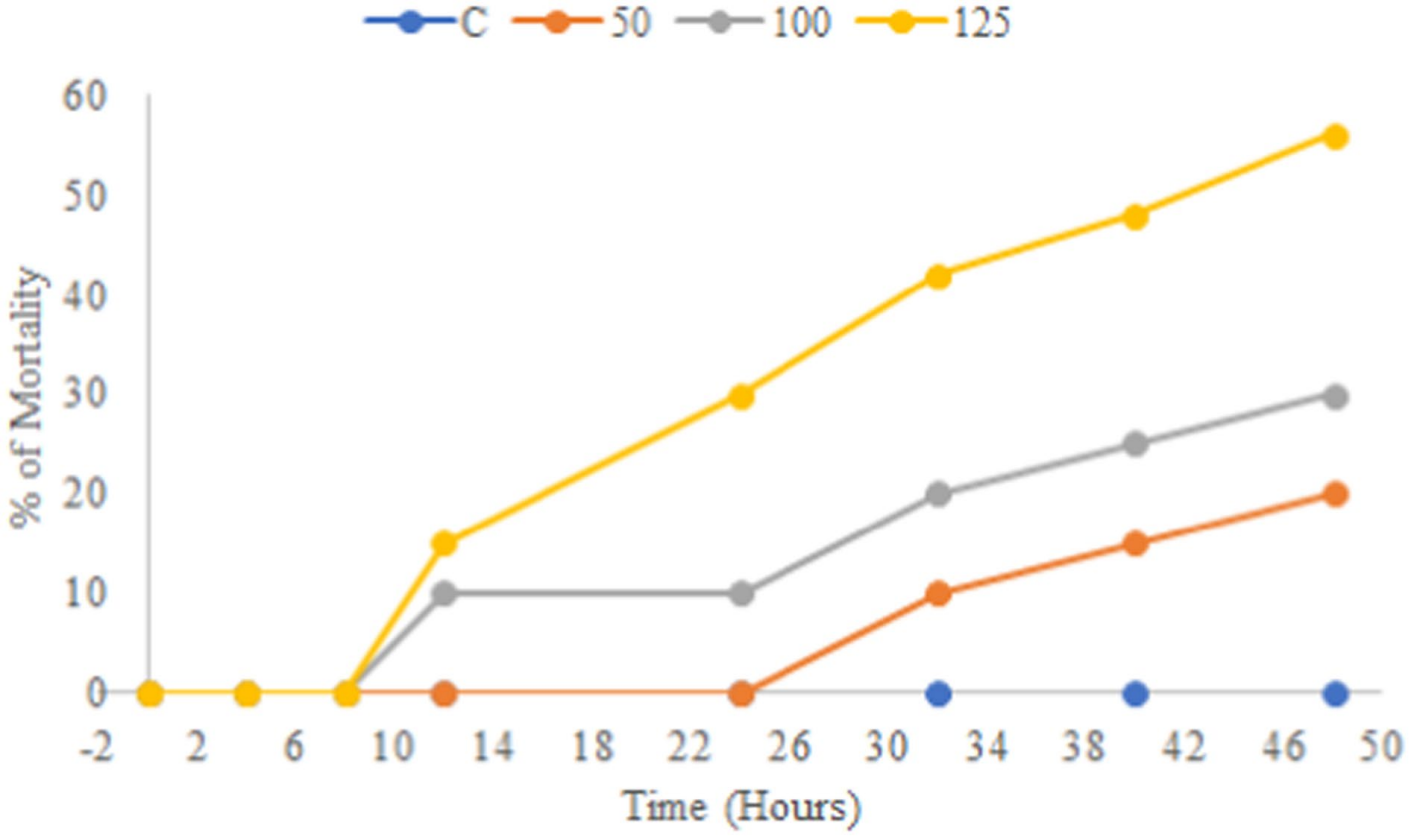
Toxicity of ethyl acetate crude extract of endophytic fungus *Paradendryphiella arenariae.*

A preliminary *in vitro* toxicity test was performed for a potent extract from an antibacterial test against bacterial fish pathogens using a brine shrimp lethality assay. The result showed that the EtOAc crude extract of endophytic fungus *Paradendryphiella arenariae* was non-toxic toward brine shrimp. All the 10 nauplii in the control were alive over a while of 48 h. The ethyl acetate extract of *Paradendryphiella arenariae* revealed a significant increase in the survival of nauplii, based on a dose-dependent manner. The results obtained from the assay are depicted in Figure 7.

### 3.9 Morphological degeneration of bacterial cells exposed to the extract

**Figure 8:**
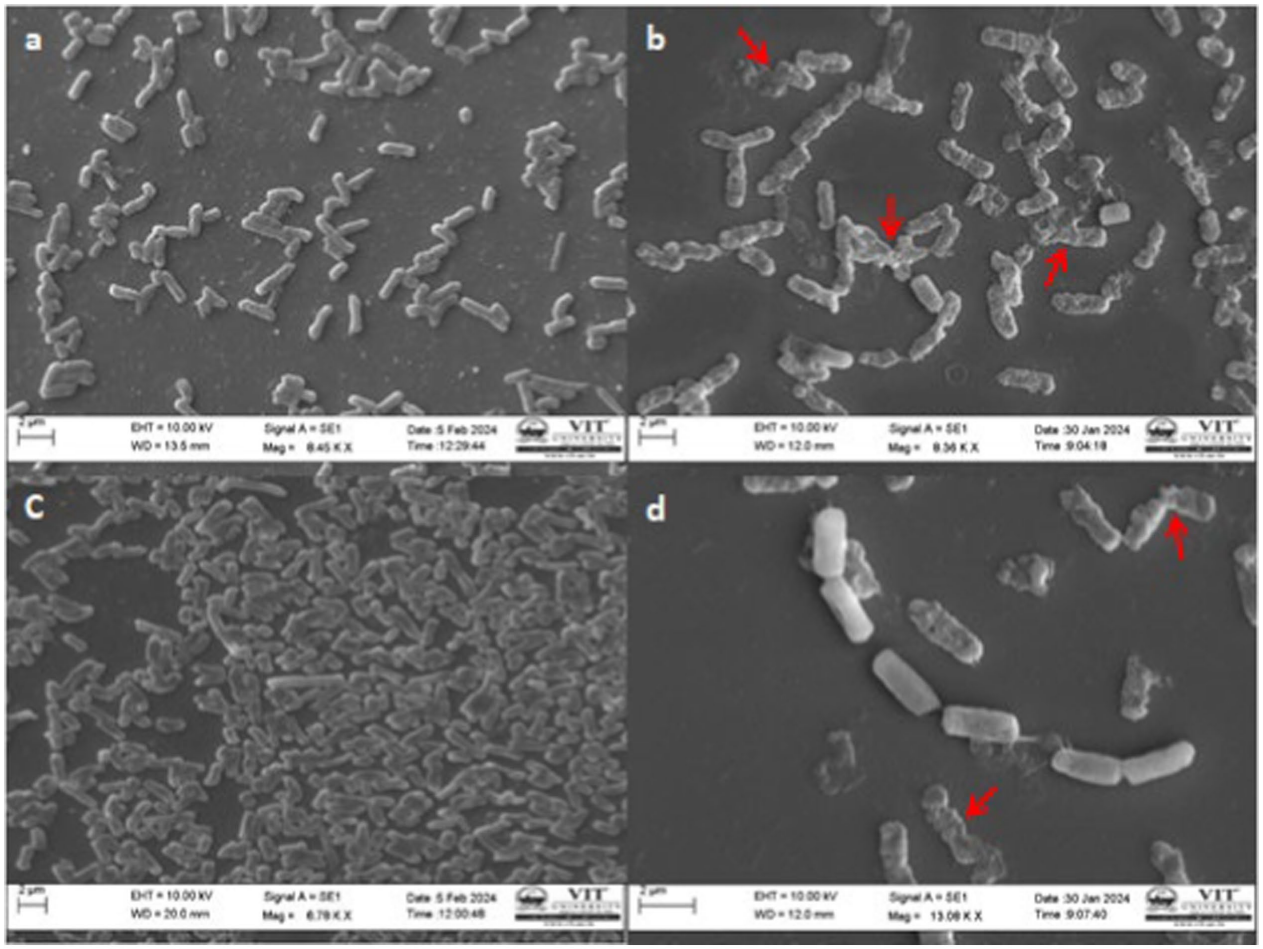
SEM Photomicrographs of the bacterial fish pathogens treated with ethyl acetate crude extract of *Paradendryphiella arenariae* at 100 μg/mL **(a)** Untreated *Aeromonas hydrophila* cell (Control), **(b)** Extract treated *A.hydrophila* cell, **(c)** Untreated *Edwardsiella tarda* cell (Control), **(d)** Extract treated *E.tarda* cell. Scale bar: 2 μm.

SEM examination indicated the effect of ethyl acetate extract on the structure and morphology of bacterial cells and this has resulted in conformity to the earlier effect of antibacterial activity. Usually, extract-treated bacterial cells were irreparable they endured morphological deterioration and were observed in SEM analysis. Photomicrographs of the Control and ethyl acetate crude extract of *Paradendryphiella arenariae* CAS1 treated *Aeromonas hydrophila* and *Edwardsiella tarda* were shown in Figure 8. The undamaged cells (Control) of *Aeromonas hydrophila* in Figure 8a illustrate without any treatment. It shows regular, rod-shaped and smooth surface bacteria. Figure 8b displays the effect of ethyl acetate crude extract treated *Aeromonas hydrophila* after 48 h of exposure resulting in the irregular shape of cells, leakage of cytoplasm cells and the surface of the cells crumpled and shrunken as compared to the normal cells. Figure 8c is the control (untreated cells) of *Edwardsiella tarda* with the presence of an intact rod-shaped and appeared to be normal. After 48 h of exposure, the ethyl acetate crude extract treated *Edwardsiella tarda* surface became rough and shrunken (indicated by the red arrow). Thus, the cells lost theircellular content and became completely disrupted are shown in Figure 8d.

### 3.10 Mycochemical assessment

The ethyl acetate extract of *Paradendryphiella arenariae* was exposed to phytochemical screening that revealed the existence of phenol, flavonoid, alkaloid, saponin and tannin. These phytoconstituents existing in the endophytic fungus are responsible for antibacterial efficiency against bacterial fish pathogens.

### 3.11 Analysis of bioactive compounds of the crude extract

**Figure 9:**
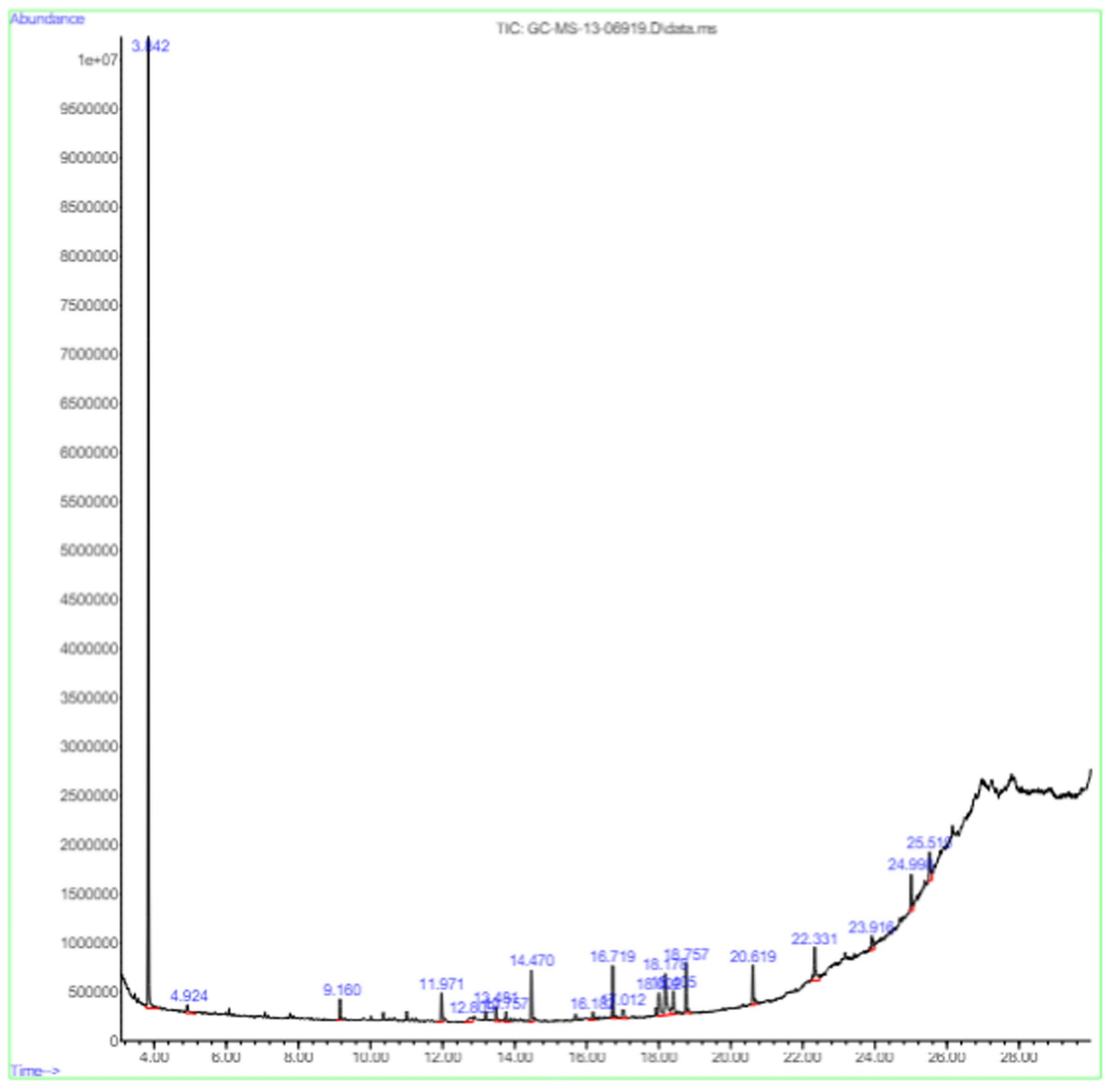
Chromatogram of ethyl acetate crude extract of *Paradendryphiella arenariae* CAS1.

The active substances present in the ethyl acetate fungal crude extract of *Paradendryphiella arenariae* CAS1 were analyzed by GC–MS. The peaks acquired in the chromatogram of GC–MS analysis were compared with the NIST database and it exhibited retention time, Molecular weight, Molecular formula and Area % of the various secondary metabolites were identified and presented in Table 4. GC–MS results confirmed the presence of major active bioactive compounds such as 4-Ethyl-3-octanol, 13-Octadecenal, (Z)-, 1-Decene, 1-Tridecene, 5,8-Decadien-2-one, 5,9-dimethyl-, (E)-, 2,4-Di-tert-butylphenol, Heptadecane, 2-methyl-, 1-Heptadecene, Eicosane, Heptafluorobutyric acid, n-tetradecyl ester, Cyclo(L-prolyl-L-valine), Hexahydro-2H-pyrido(1,2-a)pyrazin-3(4H)-one, L-Proline, N-valeryl-, undecyl ester, Dibutyl phthalate, Cycloeicosane, 1-Hexacosene, 1-Tetracosene, 1,2-Bis(trimethylsilyl)benzene, Acetamide, 2-(2,4-dimethoxybenzylidenehydrazino)-N-ethyl-2-oxo and Methyltris(trimethylsiloxy)silane. GC–MS chromatogram of ethyl acetate crude extract of endophytic fungus *Paradendryphiella arenariae* is shown in Figure 9.

**Table 4:** List of chemical compounds present in the ethyl acetate crude extract of *Paradendryphiella arenariae* CAS1.

Compound name	RT	Area	Area %	Molecular formula	Molecular weight (g/mol)	Structure
4-Ethyl-3-octanol	3.842	12,659,158	48.93	C_10_H_22_O	158.28	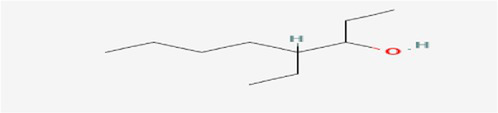
13-Octadecenal, (Z)-	4.924	322,104	1.24	C_18_H_34_O	266.5	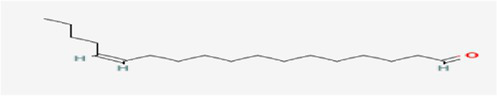
1-Decene	9.160	334,486	1.29	C_10_H_20_	140.27	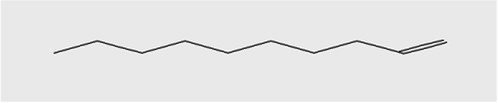
1-Tridecene	11.971	637,029	2.46	C_13_H_26_	182.35	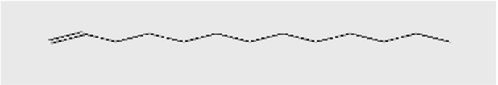
5,8-Decadien-2-one, 5,9-dimethyl-, (E)-	12.809	384,463	1.49	C_12_H_20_O	180.29	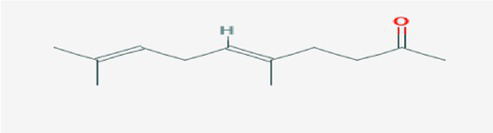
2,4-Di-tert-butylphenol	13.481	296,948	1.15	C_14_H_22_O	206.32	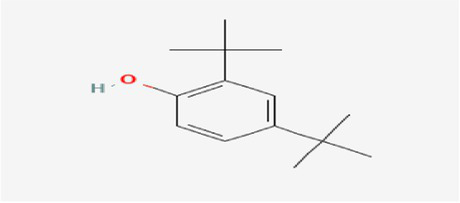
Heptadecane, 2-methyl-	13.757	280,145	1.08	C_18_H_38_	254.5	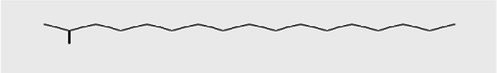
1-Heptadecene	14.470	998,161	3.86	C_17_H_34_	238.5	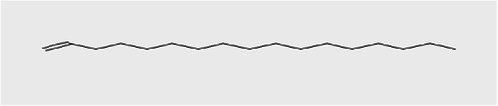
Eicosane	16.182	253,206	0.98	C_20_H_42_	282.5	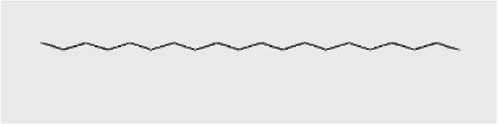
Heptafluorobutyric acid, n-tetradecyl ester	16.719	1,041,770	4.03	C_18_H_29_F_7_O_2_	410.4	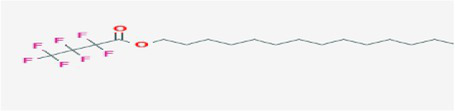
Cyclo(L-prolyl-L-valine)	17.012	370,754	1.43	C_10_H_16_N_2_O_2_	196.2	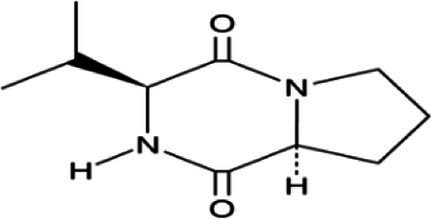
Hexahydro-2H-pyrido(1,2-a)pyrazin-3(4H)-one	18.002	895,377	3.46	C_8_H_14_N_2_O	154.21	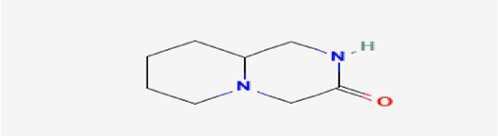
L-Proline, N-valeryl-, undecyl ester	18.178	1,692,243	6.54	C_21_H_39_NO_3_	353.5	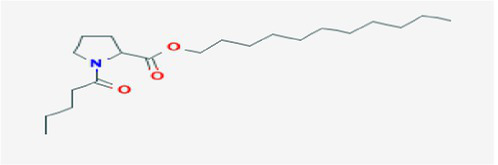
Dibutyl phthalate	18.405	711,560	2.75	C_16_H_22_O_4_	278.34	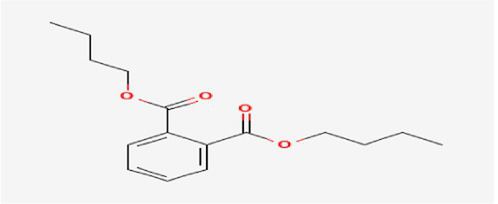
Cycloeicosane	18.757	925,839	3.58	C_20_H_40_	2800.5	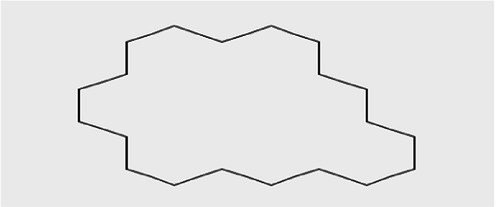
1-Hexacosene	20.619	824,998	3.19	C_26_H_52_	364.7	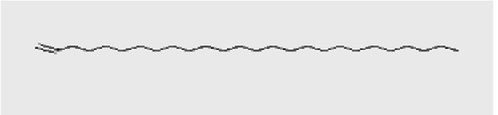
1-Tetracosene	22.331	1,144,206	4.42	C_24_H_48_	336.6	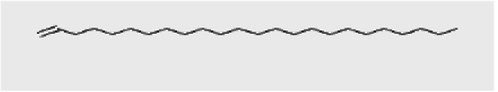
1,2-Bis(trimethylsilyl)benzene	23.916	518,312	2.00	C_12_H_22_Si_2_	222.47	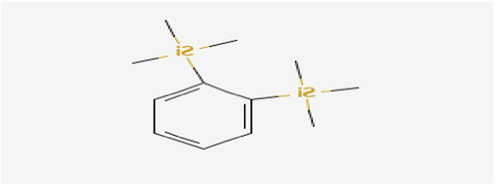
Acetamide, 2-(2,4-dimethoxybenzylidenehydrazino)-N-ethyl-2-oxo	24.998	819,317	3.17	C_13_H_17_N_3_O_4_	279.29	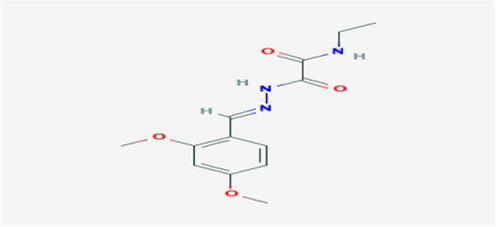
Methyltris(trimethylsiloxy)silane	25.510	763,107	2.95	C_10_H_30_O_3_Si_4_	310.68	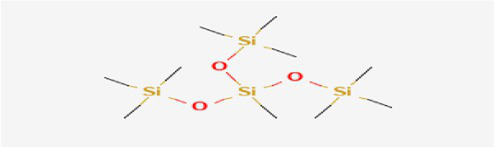

### 3.12 Fourier transform infrared spectroscopy of the fungal crude extract

**Figure 10:**
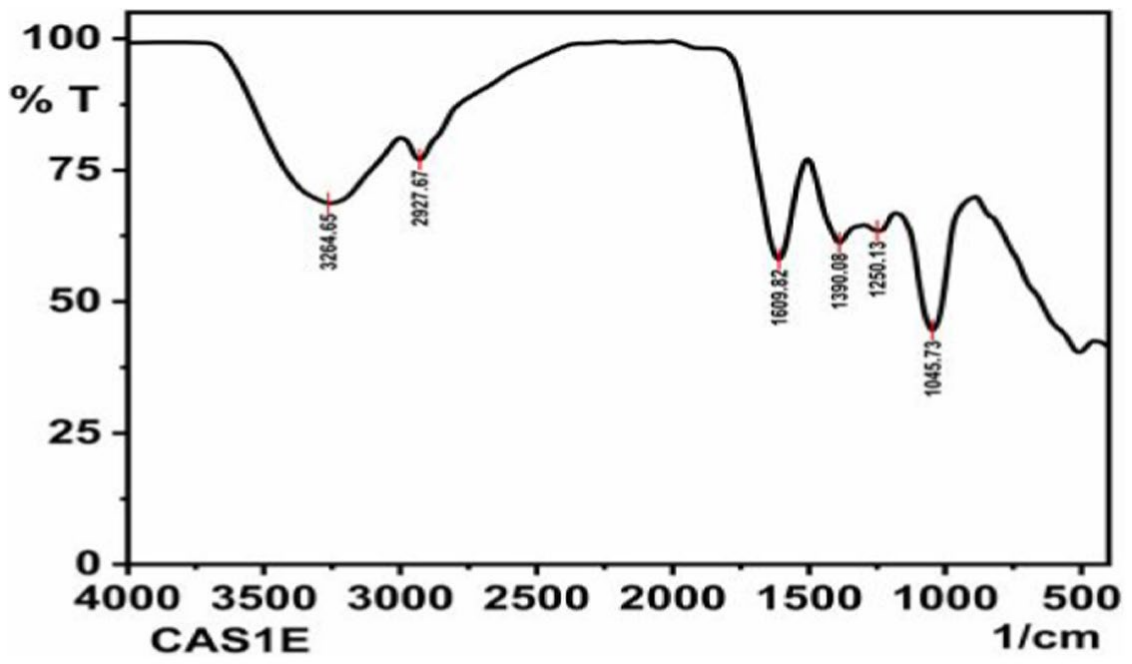
FTIR spectrum of *Paradendryphiella arenariae* ethyl acetate extract.

FTIR was performed to analyze the presence of functional groups in the ethyl acetate fungal extract of *P.arenariae* CAS1. Based on its peak ratio, the functional group of the components was separated in the FTIR. The results of FTIR spectral analysis of CAS1 showed the major peak with an intensity of 3264.65 cm^−1^ indicating the presence of NH or OH stretching vibration on the amine or alcohol group. The peak at 2927.67 cm^−1^ refers to the presence of the aromatic (the C-H bending vibration) group. The peak at 1609.02 cm^−1^ revealed C=C stretching vibration. A peak at 1390.08 cm^−1^ showed O-H bending vibration and 1250.13 cm^−1^ (C=N stretching vibration). A peak at 1045.73 cm^−1^ denotes C-O stretching vibration. FTIR Spectrum of *Paradendryphiella arenariae* CAS1 is shown in Figure 10.

## 4 Discussion

The occurrence of life-threatening, multi-drug resistant fish bacteria has urged exploring for new biologically active compounds with enormous antimicrobial properties. Medicinal plants serve as a distant environment for various endophytic microorganisms. Most of the previous studies suggested that the endophytic fungi isolated from medicinal plants have novel bioactive compounds that attenuate the growth of pathogenic bacteria. Therefore, the current study investigates the endophytic fungi for a wide range of antibacterial activity against bacterial fish pathogens. Fungal endophytes are microbes that dwell within wholesome plant tissues with no negative effects on their hosts (Gunasekaran et al., 2017). Antagonism, continuum mutualism, and neutralism were some of the interactions between host fungi during the evolutionary process. This relationship helps the endophytes by genomic background, and natural habitat and benefits host plants through increased uptake of nutrients, pathogen resistance, growth maintenance, and accrue bioactive substances (Jia et al., 2016).

Septiana et al. (2017) reported that little research was explored on the antimicrobial properties of endophytic fungi isolated from medicinal plants against fish pathogens. The preliminary antibacterial activity of endophytic fungi isolated from the stem of *Centella asiatica* revealed that the endophytic fungus *Paradendryphiella arenariae* CAS1 isolates exhibited significant antagonistic activity against selected trial pathogens. The highest antibacterial activity was observed against *Edwardsiella tarda* and *Aeromonas hydrophila* compared to *Vibrio anguillarum*. This qualitative screening decides the existence of antibacterial constituents secreted via fungal agar plugs (Taufiq and Darah, 2019).

The antagonistic activity of fermentative broth, an agar well diffusion assay was carried out for the active endophytic fungus *Paradendryphiella arenariae* CAS1 extracts against test bacteria. The results showed maximum zone of inhibitions was noted in the ethyl acetate extract compared to other tested extracts. It possesses significant antagonistic action against the tested pathogens such as *Aeromonas hydrophila* (21 ± 0.11 mm), *Aeromonas caviae* (18 ± 0.1 mm), *Edwardsiella tarda* (23 ± 0.11 mm), *Vibrio anguillarum* (19 ± 0.05 mm) and *Vibrio harveyi* (20 ± 0.27 mm) with a concentration of 100 μg/mL. The synergic effect of various biologically active substances in the ethyl acetate crude might be the reason for antagonistic action. The endophytic fungus *Cladosporium anthropophilum*, isolated from *Avicennia marina*, exhibited notable antagonistic activity against *Vibrio harveyi*, producing a zone of inhibition measuring 21.80 ± 0.26 mm. Additionally, its MIC value was determined to be 31.25 ± 0.39 g/mL (Mulyani et al., 2023). Enany et al. (2022) reported that the extracellular metabolites extracted from *Cladosporium* sp. (no 2), *Penicillium* sp., *Cladosporium* sp. (no 5), *Cladosporium* sp. (no 6), *Fusarium* sp., and *Cladosporium* sp. (no 7) isolated from *O. europaea* L. exhibited antibacterial activity against *Aeromonas hydrophila*. The Endophytic fungi *Arthrobotrys foliicola and Fusarium verticillioides*, isolated from the medicinal plant *Curcuma longa* L. displayed substantial antibacterial effect against *Morganella morganii*, a common histamine-producing bacteria found in fish, with inhibition zones 8 mm and 9.3 mm, respectively (Septiana et al., 2017). The fungal endophyte *Chaetomium* sp., isolated from the root of *Mentha piperita* L., showed significant antibacterial activity against *P. aeruginosa*, with a 12 mm zone of inhibition and a minimum inhibitory concentration (MIC) of 12.5 mg/mL (Enany et al., 2022). The compound 2-hydroxy-6-methylbenzoic acid, obtained from the endophytic fungus *Phoma* sp. associated with *Taraxacum mongolicum,* demonstrated strong antagonistic action against bacterial fish pathogens, including *Aeromonas hydrophila* and *Edwardsiella tarda* (Zhang et al., 2013). The ethyl acetate extract of *Alternaria tenuissima* EN-192, an endophytic fungus isolated from the stems of the marine mangrove plant *Rhizophora stylosa*, produced the compound Djalonensone. This compound showed moderate antibacterial activity against *Vibrio anguillarum,* with a 9 mm zone of inhibition at a concentration of 100 μg/disk (Sun et al., 2013).

Goutam et al. (2016) reported that benzyl benzoate derived from ethyl acetate of endophytic fungus *Emericella qaudrilineata* (RS-5) of medicinal plant *Pteris pellucida* demonstrated significant antibacterial activity with inhibitions zone 15 mm against *Aeromonas hydrophila*. Similarly, marine *Burkholderia cepacia* produced the compound 1.2-benzene dicarboxylic acid, (2-ethylhexyl) ester displayed efficient antagonism against *A.hydrophila*, *E.tarda* and *V.ordalii* (Verma et al., 2014). Rukisah et al. (2019) reported that prominent antibacterial efficacy was perceived in the methanol leaf extract of *Centella asiatica* with inhibition zone 10.57 mm against *A. hydrophila* and 21.14 mm against *V. harveyi* and it also contains the phytoconstituents such as alkaloid, phenol, and tannin. The crude ethyl acetate extract of Trichoderma NPK2 displayed maximum antagonistic action against *Vibrio harveyi* with a 20 mm zone of inhibitions in 200 μg/mL concentration (Narendran and Kathiresan, 2016). In the antibacterial activity, the extract concentration plays a major role in inhibiting the growth of pathogenic bacteria. The antibiotic was resistant to the bacteria when the concentration of the antibiotic agent became low, resulting in inadequate inhibitory activity (Jalil et al., 2022). The chloroform crude extract of *Centella asiatica* leaves inhibited *Edwardsiella tarda* with a zone of inhibition of 11.25 mm at 10 μL concentration (Purkait et al., 2018). According to our knowledge, no reports exist on the antagonistic activity of endophytic fungus *Paradendryphiella arenariae* extracts against bacterial fish pathogens.

The MIC and MBC values of the ethyl acetate crude extract of the endophytic fungus *Paradendryphiella arenariae* were examined by the two-fold broth microdilution method. The result revealed that the maximum MIC values of 12.5 ± 0.04 μg/mL and 25 ± 0.01 μg/mL were observed in *Edwardsiella tarda* and *Aeromonas hydrophila*. While its MBC value was attained to be 25 ± 0.5 μg/mL and 50 ± 0.2 μg/mL. If the MBC/MIC ratio was less than equal to 4, The antimicrobial compounds possess bactericidal influence toward trial microorganisms. Whereas, If the MBC/MIC ratio was greater than 4 indicates that the compounds exhibited the effect of bacteriostatic against trial microorganisms. In the broth dilution assay, the maximum growth of inhibition was noted in the *C.asiatica* chloroform extract (0–10% per mL) against *Edwardsiella tarda* (Purkait et al., 2018). The antagonistic effect of the aqueous extract from *Centella asiatica* plant revealed MIC and MBC values as 50 and 100 mg/mL^−1^, respectively, toward *Aeromonas hydrophila* (Kanchan et al., 2019). According to Ibrahim et al. (2023) the methanol extract of *Centella asiatica* at 100 mg/mL confirmed substantial antagonism with a 17 mm zone of inhibitions and also exhibited the lowest MIC and MBC values of 0.79 mg/mL and 12.50 mg/mL, respectively against *Vibrio alginolyticus*.

Besides antibacterial efficacy, *Centella asiatica* plant exhibits significant anticancer, anti-inflammatory, antioxidant, antifungal, wound healing and neuroprotective properties that can be used in aquaculture to treat various diseases (Agme-Ghodke et al., 2016). Furtado et al. (2019) suggested that *Paradendryphiella arenaria* was the only species habitually existing in the root part in a variety of samples. Our present research is the first report stating that *Paradendryphiella arenaria*, a fungal endophytes were isolated from the stem of *Centella asiastica*. The marine *Paradendryphiella* sp. exhibited maximum impeding proliferation on cancer cell lines of A549 (372.37 μg/mL), MDA-MB 231 (417.48 μg/mL) and HepG-2 (365.00 μg/mL) cells (El-Shall et al., 2023) *α*-hydroxy *γ*-butenolides, a novel compound yielded by *Paradendryphiella salina* that was connected with QS quenching of pathogenic bacteria *Pseudomonas aeruginosa* (Vallet et al., 2020). Dezaire et al. (2016) reported that *Paradendryphiella arenaria*, a marine fungus was effective on MCF7 epithelial cancer cell lines with an IC50 of 1 μg/mL and also ability to inhibit MCF7-Sh-WISP2 invasive cancer cell lines at 0.7 μg/mL.

The efficacy of CAS1 ethyl acetate extract in time-kill assay revealed a substantial bacterial cell reduction after 24 h of incubation at MIC and 2 × MIC concentrations against *Aeromonas hydrophila* and *Edwardsiella tarda*. Time-kill kinetics of DCM: Methanol extract ratio of *Centella asiatica* displayed dose and time-dependent kinetics against *Shigelli sonnei* (Sieberi et al., 2020). The toxicity study of Manilal et al. (2023) demonstrated that the *Centella asiatica* leaf extract at less than 1 mg/mL concentration was not toxic to *Artemia salina* nauplii. The present study also discovered that the endophytic fungal extract exhibited non-toxicity toward *Artemia salina* due to the active secondary metabolites that are produced by the endophytes. Thus, the endophytic fungal extract can be used as an antibacterial agent in the aquaculture industry. Mycochemical screening of the present study revealed the presence of phytoconstituents such as phenol, flavonoid, alkaloid, saponin and tannin in the ethyl acetate extract of *Paradendryphiella arenariae* which might be responsible for antibacterial inhibitions against aquaculture pathogens. Similarly, the petroleum ether and ethyl acetate extract of *centella asiatica* plant unveiled the presence of tannin, alkaloid, terpenoid and cardiac glycosides (Selvakumari and Anitha, 2016). The qualitative screening of phytochemicals in the methanolic leaf extract of *C.asiatica* indicated the existence of phenolic compounds, alkaloids, terpenoids, flavonoids, etc., (Saranya et al., 2017).

The methanolic leaf extract of *Bidens pilosa* produced the compound 13-Octadecenal, (Z)- which exhibits antimicrobial properties (Ajanaku et al., 2018). 1-Tridecene is a plant-metabolized fatty acid that possesses significant antibacterial activity (Shettar et al., 2017). The active metabolite 2,4-Di-tert-butylphenol extracted from ethyl acetate extract of *Streptomyces* sp. and displayed potential antibacterial efficacy with the MIC value 0.78 μg/mL at 50 μg/mL against *E. coli* (ATCC 25922) and *S. aureus* (ATCC 29213) and it also revealed substantial anti-proliferative activity against MCF7 (breast cancer) cell line with IC50 value 11.0 μg/mL and normal VERO cell line (IC50 Value: 116.8 μg/mL; Seenivasan et al., 2022). Cyclo(L-prolyl-L-valine) has been reported with antagonistic activity (Khedr et al., 2015). The bioactive compound Dibutyl phthalate was isolated from *Begonia malabarica* Lam. belonging to Begoniaceae family showed remarkable antagonistic effect with 9 mm inhibition zone against *S.epidermidis, E.coli, V.cholera, M.luteus, S.pneumoniae, K.pneumoniae, S.flexneri* and *P.aeruginosa at 100* mg mL^1^ (Shobi and Viswanathan, 2018). 1-Heptadecene has been stated for its antibacterial activity (Lakshmi and Nair, 2017) Eicosane, a long chain fatty acid (Faridha Begum et al., 2016) was recorded as antimicrobial agent. 1-Decene from *Tussilago farfara* L. exhibits antibacterial effectiveness against *Escherichia coli* (Boucher et al., 2020).

In the present investigation, the fungal crude extract treated *Aeromonas hydrophila* and *Edwardsiella tarda* underwent structural and morphological changes to bacterial cells compared to normal cells and was examined using the SEM. The result revealed that the cells underwent damage to the surface, shrunken and crumpled phenomenon, uneven shape of the bacteria, and leakage of cell cytoplasm. This was due to the efficacy of extract toward analyzed bacteria. Yan et al. (2023) reported that the compound Neoechinulin B obtained from *Aspergillus chevalieri* extract induces serious structural damage to the *Aeromonas hydrophila* cells that results in bacteriolysis and cell death beyond repair. *Vibrio alginolyticus* treated with different concentrations of methanol extract demonstrated roughening and shrinkage in the cell surface, breach in the cell wall and cell membrane resulted in cell lysis with expulsion of cell substances (Ibrahim et al., 2023). According to Pratama et al. (2023), the active compounds such as tannins, alkaloids, terpenoids and flavonoids existing in the extract of bitter leaf *Andrographis paniculata* treated *Edwardsiella tarda*, resulting in morphology destruction such that it clutters the metabolism of bacteria. Therefore, the active compounds present in the fungal crude extracts rupture the cell membrane, and disrupt its integrity causing the cell to shrink, cavity development, a burst of cell, and leakage of cellular materials that result in irreversible cell death.

## 5 Conclusion

The endophytic fungal isolates from *Centella asiatica* have not been explored against bacterial fish pathogens. It is the first report to isolate the endophytic fungus, *Paradendryphiella arenariae* from *Centella asiatica* stem. This is the foremost report of *Paradendryphiella arenariae*, a fungal endophyte extract that attenuates the growth of fish bacterial pathogens. In conclusion, the bioactive compounds present in the endophytic fungal extract obtained from *Centella asiatica* can be used as natural effective antibacterial agents in aquaculture. Further, the purification and identification of compounds that are responsible for inhibiting the growth of fish bacterial pathogens is yet to be done and the exact mechanism of inhibition needs to be explored.

## Data Availability

The datasets presented in this study can be found in online repositories. The names of the repository/repositories and accession number(s) can be found in the article/supplementary material.
